# Metabolic biomarkers for response to PI3K inhibition in basal-like breast cancer

**DOI:** 10.1186/bcr3391

**Published:** 2013-02-28

**Authors:** Siver A Moestue, Cornelia G Dam, Saurabh S Gorad, Alexandr Kristian, Anna Bofin, Gunhild M Mælandsmo, Olav Engebråten, Ingrid S Gribbestad, Geir Bjørkøy

**Affiliations:** 1 MI Lab, Department of Circulation and Medical Imaging, Norwegian University of Science and Technology (NTNU), PO Box 8905, N-7491 Trondheim, Norway; 2 St. Olavs University Hospital, PO Box 3250, Sluppen, N-7006 Trondheim, Norway; 3 Department of Laboratory Medicine, Children's and Women's Health, Norwegian University of Science and Technology (NTNU), PO Box 8905, N-7491 Trondheim, Norway; 4 Department of Tumor Biology, Institute for Cancer Research, Oslo University Hospital HF - Radiumhospitalet, Montebello, N-0310 Oslo, Norway; 5 Department of Pharmacy, Faculty of Health Sciences, University of Tromsø, N-9037 Tromsø, Norway; 6 Department of Oncology, Oslo University Hospital HF - Ullevaal, and Institute for Clinical Medicine, University of Oslo (UiO), PO Box 1171, Blindern, N-0318 Oslo, Norway; 7 Department of Technology, University College of Sør-Trøndelag (HiST), PO Box 2320, N-7004 Trondheim, Norway

## Abstract

**Introduction:**

The phosphatidylinositol 3-kinase (PI3K) pathway is frequently activated in cancer cells through numerous mutations and epigenetic changes. The recent development of inhibitors targeting different components of the PI3K pathway may represent a valuable treatment alternative. However, predicting efficacy of these drugs is challenging, and methods for therapy monitoring are needed. Basal-like breast cancer (BLBC) is an aggressive breast cancer subtype, frequently associated with PI3K pathway activation. The objectives of this study were to quantify the PI3K pathway activity in tissue sections from xenografts representing basal-like and luminal-like breast cancer before and immediately after treatment with PI3K inhibitors, and to identify metabolic biomarkers for treatment response.

**Methods:**

Tumor-bearing animals (*n *= 8 per treatment group) received MK-2206 (120 mg/kg/day) or BEZ235 (50 mg/kg/day) for 3 days. Activity in the PI3K/Akt/mammalian target of rapamycin pathway in xenografts and human biopsies was evaluated using a novel method for semiquantitative assessment of Akt^ser473 ^phosphorylation. Metabolic changes were assessed by *ex vivo *high-resolution magic angle spinning magnetic resonance spectroscopy.

**Results:**

Using a novel dual near-infrared immunofluorescent imaging method, basal-like xenografts had a 4.5-fold higher baseline level of pAkt^ser473 ^than luminal-like xenografts. Following treatment, basal-like xenografts demonstrated reduced levels of pAkt^ser473 ^and decreased proliferation. This correlated with metabolic changes, as both MK-2206 and BEZ235 reduced lactate concentration and increased phosphocholine concentration in the basal-like tumors. BEZ235 also caused increased glucose and glycerophosphocholine concentrations. No response to treatment or change in metabolic profile was seen in luminal-like xenografts. Analyzing tumor sections from five patients with BLBC demonstrated that two of these patients had an elevated pAkt^ser473 ^level.

**Conclusion:**

The activity of the PI3K pathway can be determined in tissue sections by quantitative imaging using an antibody towards pAkt^ser473^. Long-term treatment with MK-2206 or BEZ235 resulted in significant growth inhibition in basal-like, but not luminal-like, xenografts. This indicates that PI3K inhibitors may have selective efficacy in basal-like breast cancer with increased PI3K signaling, and identifies lactate, phosphocholine and glycerophosphocholine as potential metabolic biomarkers for early therapy monitoring. In human biopsies, variable pAkt^ser473 ^levels were observed, suggesting heterogeneous PI3K signaling activity in BLBC.

## Introduction

Basal-like breast cancer (BLBC) accounts for approximately 15-20% of breast cancers, and has the least favorable prognosis of all breast cancer subtypes. BLBC often occurs in women younger than 40 years and is associated with short time to metastasis and short overall survival compared with other subtypes of breast cancer [1,2]. Introduction of drugs targeting oncogenic signaling pathways may represent a new paradigm in the treatment of BLBC [1,3]. Basal-like breast cancer frequently exhibits the triple negative phenotype. In contrast to other breast cancer subtypes, these patients currently lack targeted treatment alternatives and would therefore benefit from the introduction of new, molecularly targeted drugs. However, introduction of targeted therapy will also depend on the development of diagnostic approaches to evaluate whether the relevant target is driving tumor progression.

For breast cancer, the presence of human epidermal growth factor receptor 2 (HER2) amplification predicts possible positive effects of injected neutralizing antibodies [4]. Predicting efficacies of a targeted drug from DNA sequence variations have proven useful for treatment of lung cancers with epidermal growth factor receptor inhibitors [5,6]. However, predicting the activity in the phosphatidylinositol 3-kinase (PI3K)/Akt/ mammalian target of rapamycin (mTOR) pathway based on DNA sequence alterations is complex. The activity in the pathway seems to depend on a number of alternative mechanisms, including amplification or activating mutations in *PIK3CA*, loss of phosphatase and tensin homolog (PTEN) protein at a DNA, mRNA or protein level, or activating mutations/amplification in *AKT1/AKT2 *[7-10]. Owing to the number of different mechanisms that, directly or indirectly and at different levels, can lead to elevated PI3K pathway activity, development of methods that quantitatively report on signaling activity in the tumor tissue is tempting. Conventional immunohistochemistry using antibodies for active, phosphorylated Akt has been suggested, but this approach is limited by its low linear range and by the difficulty in introducing a second stain for normalizing purposes.

To accelerate the introduction of targeted drugs into clinical practice, identification of molecular biomarkers for early monitoring of response to therapy and development of resistance is required [11,12]. Assessment of tumor metabolism using magnetic resonance spectroscopy (MRS) is a promising approach for biomarker discovery, since the metabolic characteristics of cancer are inherently different from normal tissue and since oncogenic signaling regulates energy metabolism in cancer cells [13,14]. Identification of metabolic biomarkers is therefore an important step in the introduction of rational, personalized treatment of BLBC patients with drugs targeting oncogenic signaling.

Inhibitors targeting components of the PI3K pathway are a promising new class of drugs currently evaluated in various cancers. They are of particular interest in BLBC, because abnormal activity in the PI3K/Akt/mTOR signaling axis has been described both in preclinical models and in clinical cohorts in this breast cancer subtype [8,15-17]. Metabolic effects of PI3K inhibition in cancer have been studied *in vitro *and *in vivo *[12]. However, data on metabolic effects in basal-like breast cancer are lacking, and the effect of PI3K inhibition on choline metabolism in breast cancer has not yet been studied in *in vivo *models. Different subtypes of cancer have distinct metabolic profiles and the flux through metabolic pathways is in part governed by the oncogenic signaling.

We have therefore studied PI3K/mTOR/Akt pathway activity in basal-like and luminal-like breast cancer xenografts, and the effect of the pan-Akt inhibitor MK-2206 and the dual PI3K/mTOR inhibitor BEZ235 in these models *in vivo*. The response to treatment was evaluated both with respect to tumor volume, cellular proliferation and blockade of PI3K signaling. Metabolic changes in the tumor tissue were examined by *ex vivo *high-resolution magic angle spinning (HR MAS) MRS. The objectives of the study were to use a novel immunofluorescence imaging method to quantify the level of pAkt^ser473 ^in tumor tissue sections, to determine whether inhibition of the PI3K signaling pathway caused anti-tumor effects in the basal-like xenograft model, and to identify metabolic biomarkers associated with response to treatment.

## Materials and methods

### Animal models

The MAS98.12 (basal-like) and MAS98.06 (luminal-like) breast cancer xenograft models have previously been established by orthotopic implantation of biopsy tissues from primary mammary carcinomas in severe combined immunodeficiency mice [18].

For both xenograft models, animals were randomized into the following treatment groups (*n *= 8 per group): vehicle control (0.2 ml/day), MK-2206 (120 mg/kg/day) and BEZ235 (50 mg/kg/day). MK-2206 (Selleck Chemicals, Houston, TX, USA) was dissolved in dimethyl sulfoxide and diluted in 30% Captisol^® ^(CyDex Pharmaceuticals, Inc., Lenexa, KS, USA) to a final concentration of 15 mg/ml. BEZ235 (Selleck Chemicals) was dissolved in *N*-methyl-2-pyrrolidone and diluted in 30% Captisol^® ^to a final concentration of 6.5 mg/ml. Vehicle control solution consisted of dimethyl sulfoxide, *N*-methyl-2-pyrrolidone and 30% Captisol^® ^(1:1:2). These dose levels have previously shown efficacy in murine xenograft models [19,20].

The drugs were administered by gavage for 3 consecutive days. Tumor volume was measured before and after treatment using external calipers (volume = π*ab*^2 ^/ 6, where *a *and *b *represent the longest diameter and shortest diameter, respectively). After treatment, tumor tissue was harvested and preserved for histopathology (4% neutral buffered formalin) or snap frozen in N_2_(l) for metabolic profiling.

An additional batch of mice (*n *= 5 or 6 per group) was randomly assigned to treatment as described above when the tumor diameter reached approximately 5 mm and was treated with MK-2206 or BEZ235 for up to 26 days. The tumor volumes were measured regularly with calipers during the treatment period.

All procedures and experiments involving animals were approved by the National Animal Research Authority, and were carried out according to the European Convention for the Protection of Vertebrates used for Scientific Purposes.

### Histopathology

Tumor tissue was fixed in 4% formaldehyde immediately after isolation from the animal and embedded in paraffin. Sections were cut at 4 µm and mounted on glass slides. Immunohistochemical staining for the mitosis marker phosphohistone H3 (PHH3) was carried out as previously described [21]. Mitotic activity was counted in PHH3-stained sections according to Skaland and colleagues [22], and was reported as the number of positive counts per 10 fields of view. Necrotic areas were avoided.

For analysis of the activity in the PI3K/Akt pathway, sections were co-stained with mouse anti-pan-Akt antibody (#2920; Cell Signaling Technology, Beverly, MA, USA) and rabbit anti-pAkt^ser473 ^antibody (#4060; Cell Signaling Technology). Four different secondary antibodies were used to image binding of the primary antibodies. For confocal microscopy, anti-mouse conjugated with Alexa 488 and anti-rabbit conjugated with Alexa 555 (Invitrogen, Paisley, UK) were used. For near-infrared (NIR) immunofluorescence imaging, anti-rabbit conjugated with IR-680 dye and anti-mouse conjugated with IR-800 dye (Li-Cor Biosciences, Lincoln, NE, USA) were used. All secondary antibodies were added simultaneously in a 1:1 ratio to allow combined low-resolution quantifications using NIR fluorescent scanning and high resolution of regions of interest using confocal microscopy in the visible area of the light spectra. Negative control sections were prepared by staining with secondary antibodies only.

### Near-infrared immunofluorescence imaging

Stained tissue sections (*n *= 4 in all treatment groups) were scanned on an Odyssey Infrared Imaging System (Li-Cor Biosciences) with a spatial resolution of 21 µm. The samples were scanned simultaneously to enable quantitative image analysis. The signals were recorded in separate channels for concurrent imaging of pAkt^ser473 ^(700 nm) and total Akt (800 nm) levels. The images were exported as colorized 32-bit .tiff files and the signal intensity was quantified using ImageJ (National Institute of Health, Bethesda, MD, USA). Regions of interest enveloping the entire tumor area were defined and the mean signal intensity for each region of interest was determined. Compensation for autofluorescence and unspecific antibody binding was performed by subtraction of the mean signal from adjacent negative control sections. The signal intensity was compared across treatment groups using the Student's *t *test with the threshold for statistical significance defined as *P *≤0.05. Confocal microscopy was carried out using an Axiovert microscope (Carl Zeiss MicroImaging Inc., Jena, Germany) with 20× and 63× magnification, and images were captured and analyzed using Zeiss LSM Meta and Zeiss LSM Image Examiner (Carl Zeiss MicroImaging Inc.).

### Human breast cancer biopsies

To evaluate the feasibility of the NIR immunofluorescence imaging method in human tumor tissue, five paraffin-embedded specimens from patients with BLBC were retrieved from the Breast Cancer Subtypes research biobank, NTNU, which has been approved by the Regional Research Ethics Committee. The tumors were classified as BLBC using immunohistochemical and *in situ *hybridization methods as surrogates for gene expression profiling. On immunohistochemical stained tissue microarrays, the tumors were estrogen receptor negative (249R-16/SP1; Cell Marque, Rocklin, CA, USA) and progesterone receptor-negative (NCL-PGR 312-CE; Leica Biosystems, North Ryde, Australia) but were positive for cytokeratin 5 (Ncl-CK5-L-CE; Leica Biosystems) and/or epidermal growth factor receptor (K1494/2-18C9; Dako Denmark/Glostrup, Denmark) developed using pharmDx™ (Dako, Denmark). The tumors were also negative for HER2 using chromogenic *in situ *hybridization for the *HER2 *gene and the chromosome 17 centromere (*HER2 *CISH pharmDx™; Dako, Denmark) (gene:chromosome ratio <2.0).

For NIR fluorescence staining, the clinical samples were stained and imaged according to the protocol described above. The primary antibodies were omitted as a negative control of the immunostaining. The sections were stained, imaged and processed simultaneously and quantifications were performed using the Li-Cor software. After subtracting the signal intensity for the negative control in each channel, the mean intensity for the anti-pAkt^ser473 ^labeling was divided by the signal intensity for the total Akt labeling. One of the biopsies contained both normal and cancerous tissue and allowed comparison of the pAkt^ser473 ^signal in the different parts of the section.

### Western blotting

Snap-frozen tumor samples were thawed and immediately lysed in a lysis buffer (50 mM Tris-HCl, pH 8.0, 150 mM NaCl, 1 mM ethylenediamine tetraacetic acid, 1% NP-40, 0.25% Triton X-100) with phosphatase inhibitor (Complete Lysis-M; Roche Diagnostics, Indianapolis, IN, USA) and a combination of phosphatase inhibitor cocktails 2 and 3 (Sigma-Aldrich, St Louis, MO, USA). The protein concentration was determined in clear cell lysates and equal amounts of total protein (50 μg) were separated by SDS-PAGE. After immunoblotting, the membranes were developed using a mixture of the anti-pAkt^ser473 ^and pan-Akt antibodies and were imaged after labeling with NIR fluorescent secondary antibodies. PTEN levels in the tumor lysates were detected using a C-terminal PTEN antibody (#18-0256; Invitrogen) and pAkt^thr308 ^detected by a monoclonal rabbit antibody (#2965; Cell Signaling Technology). The amount of β-actin (#ab6276; Abcam, Cambridge, UK) in the lysates was used as control of equal protein loading. Binding of the respective primary antibodies was detected using secondary antibodies labeled with NIR fluorescent dyes. The images from the Odyssey Infrared Imaging System were processed using the Li-Cor software and mounted using Canvas (ACD Systems International Inc, Seattle, WA, USA).

### Metabolic profiling using high-resolution magic angle spinning magnetic resonance spectroscopy

Frozen xenograft tissue was cut to fit into 30 µl disposable inserts (Bruker BioSpin, Ettlingen, Germany) filled with 3 µl PBS/D_2_O buffer containing trimethylsilylpropanoic acid as a chemical shift reference. The average weight of the tissue samples was 12 ± 3 mg. Samples were analyzed using a Bruker AVANCE DRX-600 spectrometer equipped with a ^1^H/^13^C HR MAS probe (Bruker BioSpin). Samples were spun at 5 kHz and the instrument temperature was maintained at 4°C for all experiments. A single-pulse experiment (zgpr; Bruker) was performed for all samples. The water resonance was suppressed using a presaturation delay of 3 seconds and an acquisition time of 3.40 seconds. A sweep width of 16 ppm was used for signal collection. Thirty-two free induction decays were acquired into 64k points.

A creatine reference solution (9.05 μmol/g) was analyzed under identical conditions for use as an external calibration standard. Post-processing of spectra included 0.3 Hz exponential line broadening and baseline correction using a fifth-order function.

Chemical shifts were calibrated to the trimethylsilylpropanoic acid at 0.0 ppm. Assignment of metabolite peaks was performed with reference to previously published data [23]. The peak area of each metabolite was calculated by polynomial regression (PeakFit v 4.12; Systat Software Inc, Chicago, IL, USA). The correlation coefficient of the fit (*r*^2^) for all spectra was ≥0.95. Concentration of each metabolite was calculated with reference to the recorded sample weight and the peak area of the creatine reference solution. Metabolite concentrations were compared across treatment groups using Student's *t *test with the threshold for statistical significance defined as *P *≤0.05.

## Results

### Determining PI3K pathway activity in basal-like and luminal-like xenografts

Previous gene expression analysis has suggested increased PI3K signaling in the MAS98.12 xenograft model, which represents basal-like breast cancer [18]. Another model established in the same study represents estrogen receptor-positive luminal-like breast cancer, and was not associated with high PI3K signaling activity. We therefore hypothesized that a difference in PI3K/Akt/mTOR pathway activity in these two xenograft models could be detected by immunostaining.

**Figure 1:**
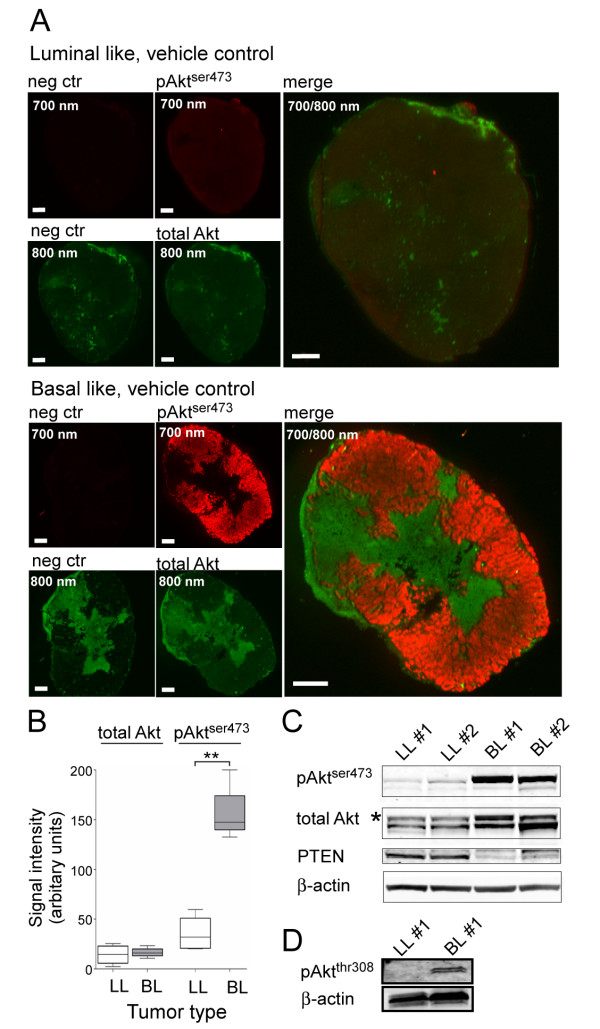
**pAkt^ser473 ^is elevated and phosphatase and tensin homolog lost in basal-like but not luminal-like xenografts**. **(A) **Immunostaining of luminal-like (LL; top) and basal-like (BL; bottom) xenograft tumor sections from the vehicle control group, stained with anti-pAkt^ser473 ^(red) and anti-pan-Akt (green) antibodies, and secondary antibodies conjugated with near-infrared (NIR) fluorescent dyes (red channel, pAkt^ser473^; green channel, pan-Akt). Negative control sections were stained with secondary antibodies alone (left two images in the panels). Tumors were isolated 3 days after the start of the treatment and 5 hours after the last supplementation of the respective drugs. All scale bars = 1 mm. **(B) **Quantification data from NIR immunofluorescence imaging (*n *= 4). The signal intensity of the total Akt immunostaining is similar in BL and LL xenografts, whereas the signal for pAkt ^ser473 ^is approximately fivefold higher in BL compared with LL xenografts. **P <0.0005. **(C) **Immunoblot analysis of the level of pAkt^ser473^, total Akt and phosphatase and tensin homolog (PTEN) in lysates from two different BL and LL cancers. Level of β-actin included as a loading control. Numbers above the images and the lanes in the immunoblot refer to the individual tumor-bearing mouse. **(D) **Higher phosphatidylinositol 3-kinase (PI3K) signaling activity in BL xenografts was also confirmed by immunoblot analysis of pAkt^thr308 ^levels.

PI3K indirectly activates the downstream kinase Akt, which is activated by phosphorylation of two sites: threonine 308 and serine 473. To determine the activity of this pathway we therefore stained for the phosphorylated activated form of Akt (pAkt^ser473^). NIR immunofluorescence imaging demonstrated a clearly increased pAkt^ser473 ^signal in the basal-like xenografts (Figure 1A). The increase was due to a specific activation of Akt since no differences in the total Akt level between the two cancer types could be observed. Omitting the primary antibodies against active and total Akt demonstrated a very low background staining in the 700 nm channel for the rabbit IgG detecting pAkt^ser473^. In the 800 nm channel used to image total Akt, however, regional staining was observed even in the absence of the primary antibody. The unspecific staining was confined to areas containing stromal tissue and to necrotic areas. This nonspecific binding of the secondary antibody most probably represents binding of the secondary anti-mouse IgG antibody to host immunoglobulins. Despite the nonspecific binding, we could still observe a specific signal of total Akt that is considered to reflect all Akt isoforms in the tumor cells. By quantification of the NIR immunofluorescence images, we corrected for nonspecific binding by subtracting the signal intensity in an adjacent tissue section. Comparing the signal intensity of stained sections from the xenografts we found a nearly fivefold higher pAkt^ser473 ^signal in basal-like tumors compared with luminal-like tumors (Figure 1B). The total Akt signal was higher than the negative control in all examined specimens (*P *<0.00003) but we found no difference in total Akt between the two tumor types (Figure 1B).

To confirm the findings from immunofluorescence imaging, the level of pAkt^ser473 ^was determined in tumor lysates by western blotting (Figure 1C). The anti-pAkt^ser473 ^antibody generated a band at the expected location. This band was eightfold increased in extracts from basal-like tumors compared with luminal-like tumors. For the quantifications, the pAkt^ser473 ^signal was normalized to the total Akt levels in the respective samples. The immunoglobulin heavy chain from the xenograft host gave an approximately 50 kDa band that migrated slightly faster than Akt, and could be detected in the absence of the primary total Akt antibody. However, the total Akt signal could only be detected after incubation with the primary antibody. Activation of Akt involves also the phosphorylation of the threonine 308 residue. However, even though an elevated level of pAkt^thr308 ^could be detected in extracts of the basal-like tumors by immunoblotting (Figure 1D), we could not obtain acceptable signal-to-noise ratios using the pAkt^thr308 ^antibody in immunofluorescence images. Previous gene expression analysis has identified a clear reduction in mRNA levels of the tumor suppressor *PTEN *in the basal-like xenograft [18]. Accordingly, the level of PTEN protein was more than six times lower in the extracts from basal-like xenografts compared with luminal-like xenografts (Figure 1C).

**Figure 2:**
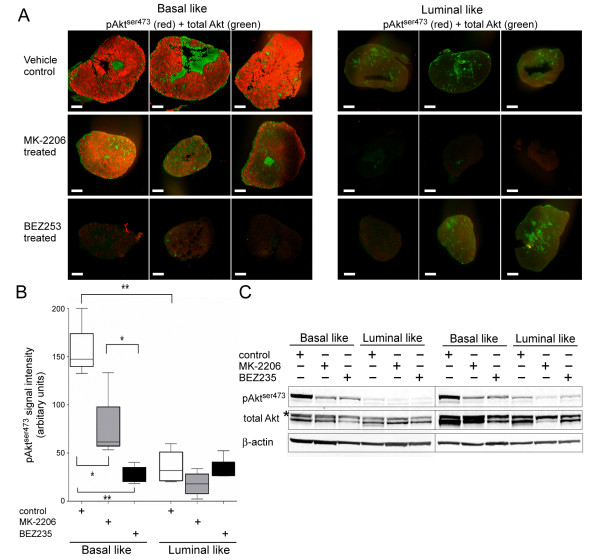
**Three-day treatment of drugs targeting phosphatidylinositol 3-kinase pathway reduces active Akt level in basal-like xenografts**. **(A) **Immunostaining of tissue sections from basal-like (BL; left) and luminal-like (LL; right) xenografts given vehicle control (upper panel), MK-2206 (middle panel) or BEZ253 (lower panel). pAkt^ser473 ^(red) and total Akt (green) are stained as in Figure 1. All scale bars = 1 mm. **(B) **Quantification data from near-infrared (NIR) immunofluorescence imaging (*n *= 4). The signal intensity was significantly reduced after treatment with MK-2206 and BEZ235 in BL but not in LL xenografts. *P <0.05, **P <0.0005. **(C) **Immunoblot analysis of tumor lysates for the level of pAkt^ser473 ^(upper panel) and β-actin loading control (lower panel). Lysates were prepared from tumors harvested 3 to 6 hours after the last administration of MK-2206 and BEZ235.

We then assayed the effect of the PI3K pathway inhibitors BEZ253 and MK-2206 on the pAkt^ser473 ^levels (Figure 2A). Immunostaining of sections from the basal-like xenografts demonstrated sixfold and twofold reductions in the pAkt^ser473 ^level in response to treatment with BEZ253 and MK-2206, respectively (*P *<0.01) (Figure 2B). Although BEZ235 had a strong inhibitory effect on the pAkt^ser473 ^level in basal-like xenografts, the observed signal was still significantly higher than in the negative control (*P *= 0.01). In the luminal-like xenografts, no significant reduction of the low level of pAkt^ser473 ^in response to any of the two compounds was observed. To verify that the differences in staining intensity were due to reduced pAkt^ser473 ^levels, we analyzed lysates from the frozen cancer samples by immunoblotting (Figure 2C). In accordance with the immunostaining, we found a clear reduction in the pAkt^ser473 ^level in the lysates from basal-like tumors in response to both MK-2206 and BEZ235. No changes in total Akt level were observed after any treatment.

**Figure 3:**
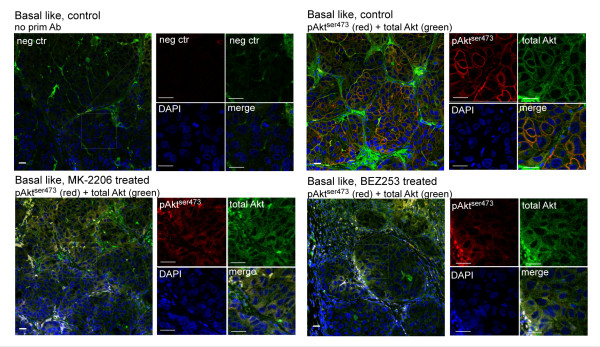
**Plasma membrane-associated pAkt^ser473 ^is lost in response to targeted inhibition of the phosphatidylinositol 3-kinase pathway**. Tumor sections from vehicle control (two upper panels) or MK-2206-treated (lower left panel) or BEZ253-treated (lower right panel) mice were stained with secondary antibodies alone (negative control; upper left panel) or with anti-pAkt^ser473 ^(red) and total Akt (green). DNA was visualized by 4',6-diamidino-2-phenylindole (DAPI) staining. Images were obtained using identical settings in the confocal microscope using a 20× objective. The sections were first imaged with a near-infrared (NIR) scanner and subsequently imaged by confocal microscopy to detect the fluorescent-labeled antibodies in the visible light area. Treatment with MK-2206 or BEZ235 causes a marked reduction of pAkt^ser473 ^levels, and loss of membrane localization compared with vehicle-treated controls. Numbers above the images refer to the individual tumor bearing mouse. All scale bars = 20 µm.

The use of NIR dyes conjugated to the respective secondary antibodies allowed co-staining with secondary antibodies that can be imaged by conventional confocal microscopy. Based on the finding that basal-like xenografts had a significantly elevated pAkt^ser473 ^level, the subcellular localization of pAkt^ser473 ^was examined by confocal microscopy. In basal-like control tumors, a clearly elevated plasma membrane-enriched pAkt^ser473 ^signal was observed. In response to treatment with MK-2206 and BEZ235, this signal was clearly reduced (Figure 3). As for the NIR scanning, we observed an unspecific signal in the 800 nm channel for total Akt that probably represents binding of anti-mouse IgG secondary antibodies to xenograft host immunoglobulins. This unspecific staining seemed to be limited to extracellular space consistent with binding of the secondary antibody to host immunoglobulins. However, there was still a detectable specific intracellular signal for total Akt that was enriched in the plasma membrane in tumors from untreated animals but more diffuse in the cytosol after treatment. No nuclear staining of pAktser473 was observed.

In summary, immunostaining of tumor sections detected either by NIR scanning or confocal microscopy as well as immunoblotting of lysates from the same tumors demonstrates that the level of pAkt^ser473 ^was elevated in the basal-like xenografts compared with the luminal-like xenografts. Further, we observed a marked reduction of pAkt^ser473 ^levels in response to treatments targeting the PI3K pathway in the basal-like xenograft model. In the luminal-like cancer model, the level of pAkt^ser473 ^was low in the vehicle-treated control animals and we could not observe any reduction of this low level in response to treatment.

### PI3K pathway activity in human basal-like breast cancer

**Figure 4:**
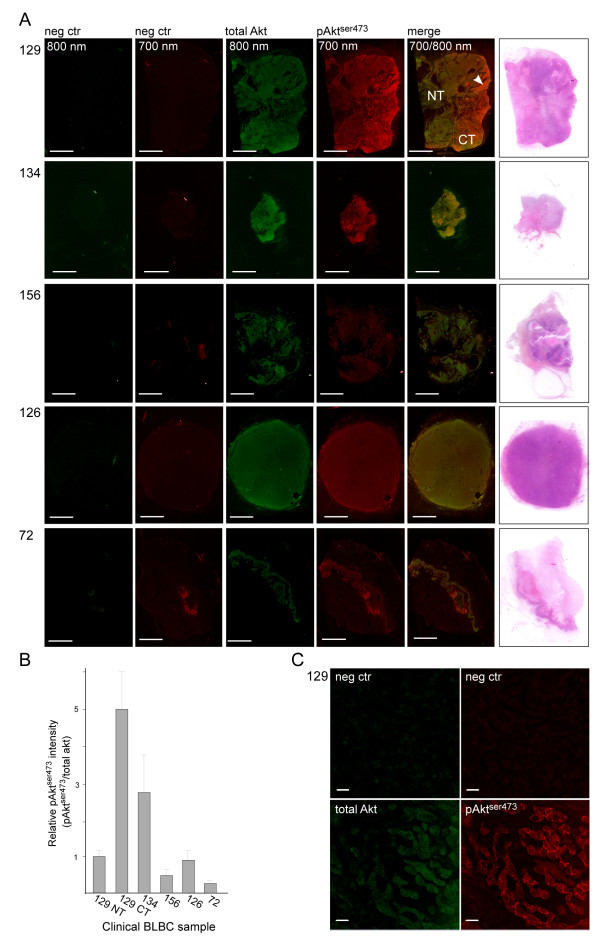
**pAkt^ser473 ^is elevated in a subset of clinical samples of basal-like breast cancer**. **(A) **Sections from five clinical samples classified as basal-like breast cancer were immunostained for total Akt (green, 800 nm) or pAkt^ser473 ^(red, 700 nm) and scanned using the near-infrared (NIR) scanner. The sample from Case 129 contains both normal tissue (NT) and cancerous tissue (CT). In Case 72, there was a longitudinal structure in the section that contained skin and resulted in a structure that is faintly visible in the negative control and also demonstrated elevated signal of both total Akt and pAkt^ser473^. The samples from the last three cases were classified as homogeneous basal-like breast cancer (BLBC). Scale bar = 5 mm. **(B) **Quantification of the pAkt^ser473 ^signal relative to the total Akt signal in the different clinical BLBC samples. For Case 129, the pAkt^ser473 ^was quantified both in the NT and in the CT. Quantifications were done in three to five randomly selected circular regions of interest of the tumors and presented as the mean relative intensity in the different areas with standard deviations. **(C) **The pAkt^ser473 ^signal is mainly located to the plasma membrane of the cancer cells in BLBC. Imaged area is from the area labeled with an arrowhead in (A). Scale bar = 20 μm.

NIR scanning and confocal microscopy demonstrated an elevated level of pAkt^ser473 ^in the MAS98.12 basal-like xenograft model. To see whether this animal model is representative for BLBC, we determined the level of pAkt^ser473 ^in tumor sections from five human BLBC biopsies. As expected, we observed that there was very little unspecific signal in the absence of the primary antibodies (Figure 4A). This highly reduced background in the clinical BLBC samples is very probably due to the absence of mouse immunoglobulins that can bind the secondary antibodies and give an unspecific signal. The sample that demonstrated strongest pAkt^ser473 ^signal contained both normal and cancerous tissue. Importantly, the pAkt^ser473 ^signal was found elevated only in the part of the sample that contained tumor cells.

The pAkt^ser473 ^signal was then quantified relative to total Akt. The cancerous tissue in the heterogeneous section had an approximately five times higher pAkt^ser473 ^level compared with the normal tissue in the same section (Figure 4B). Another of the samples also displayed an elevated pAkt^ser473 ^signal, but this sample also contained more total Akt. This sample from the pathologist was classified as homogeneous cancerous tissue and was found to have an approximately threefold higher pAkt^ser473 ^signal after normalization against the total Akt staining. The last three BLBC samples did not show elevated pAkt^ser473 ^levels. In line with the basal-like xenograft model, we found that pAkt^ser473 ^was mainly located to the plasma membrane in the cancerous tissue of the section that demonstrated a fivefold increase in pAkt. None of the other samples demonstrated a pAkt signal that could be detected using the confocal microscope.

Collectively, these results suggests that the established NIR-based immunofluorescence protocol for semiquantitative dual imaging of pAkt^ser473 ^and total Akt is well suited for analysis of clinical samples. Moreover, the five clinical samples analyzed confirm the variability in PI3K signaling observed in other studies of BLBC [8,24].

### *In vivo *anti-tumor effects of PI3K inhibitors in basal-like and luminal-like xenografts

There was a marked difference in response to PI3K inhibition between the two xenograft models. The volume of basal-like xenografts treated with MK-2206 or BEZ235 was reduced 3 days after initiation of treatment (-11 ± 26% and -12 ± 17%, respectively, compared with the pretreatment volume). In contrast, the volume of vehicle-treated basal-like xenografts increased significantly (28 ± 30% from the pretreatment volume, *P *= 0.002) in the same timespan. In luminal-like xenografts, no significant change in tumor volume was observed either in controls or treated animals. The absence of volume change in luminal-like xenografts over the 3-day treatment course could, however, reflect the slow growth rate of this model.

**Figure 5:**
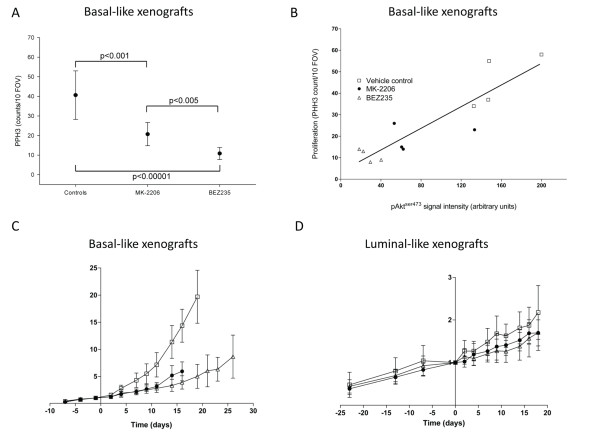
**Reduced pAkt^ser473 ^levels are correlated with tumor growth inhibition and reduced proliferation in basal-like xenografts**. **(A) **Mitotic activity (phosphohistone H3 (PHH3) counts/10 fields of view) in basal-like and luminal-like xenografts after treatment with MK-2206 and BEZ235. **(B) **Correlation between pAkt^ser473 ^signal intensity and mitosis in basal-like xenografts treated with vehicle (open squares), MK-2206 (filled circles) and BEZ235 (open triangles). **(C) **Tumor volumes (relative to day 0) in basal-like xenografts treated with MK-2206 (filled circles) and BEZ235 (open triangles), compared with vehicle-treated controls (open squares). **(D) **Tumor volumes (relative to day 0) in luminal-like xenografts treated with MK-2206 (filled circles) and BEZ235 (open triangles), compared with vehicle-treated controls (open squares).

In the vehicle-treated controls, mitotic activity (measured by PHH3 staining) was higher in basal-like xenografts than in luminal-like xenografts (41 ± 12 vs. 26 ± 4 counts/10 fields of view, *P *<0.01). This increased activity confirms the faster growth rate of the basal-like xenografts. In the basal-like xenografts, PI3K inhibition significantly (*P *<0.005) reduced the mitotic activity (21 ± 6 and 11 ± 3 counts/10 fields of view) for MK-2206 and BEZ235, respectively) (Figure 5A). The reduction in mitotic activity in the BEZ235 group was stronger than in the MK-2206 group (*P *<0.005). In the luminal-like xenografts, BEZ235 treatment did not reduce the mitotic activity (18 ± 10 counts/10 fields of view). In the MK-2206 group, a paradoxical increase in mitotic activity was observed (*P *<0.001). The reduction in pAkt^ser473 ^in basal-like xenografts treated with BEZ235 and MK-2206 correlated strongly with the mitotic rate (Figure 5B).

Long-term treatment with MK-2206 and BEZ235 caused a significant growth delay in basal-like xenografts (Figure 5C). At the time point where vehicle-treated controls had to be sacrificed due to their tumor burden (19 days after start of treatment), the tumor volume of BEZ235-treated mice was 33% of the controls (*P *<0.00001). No significant difference between BEZ235-treated and MK-2206-treated mice was observed. In the slower-growing luminal-like xenografts, there was no significant difference between the treated group and the vehicle control group (*P *= 0.14) (Figure 5D).

### Identification of metabolic biomarkers for response to PI3K inhibition

The metabolic profiles from vehicle-treated tumors confirmed the differences between basal-like and luminal-like xenografts observed in previous studies [25,26]. This model has a characteristic metabolic profile, with a glycerophosphocholine (GPC):phosphocholine (PCho) ratio >1 and significantly higher glycine concentration than the luminal-like xenograft. The metabolite concentrations from all treatment groups are presented in Table S1 in Additional file 1.

**Figure 6:**
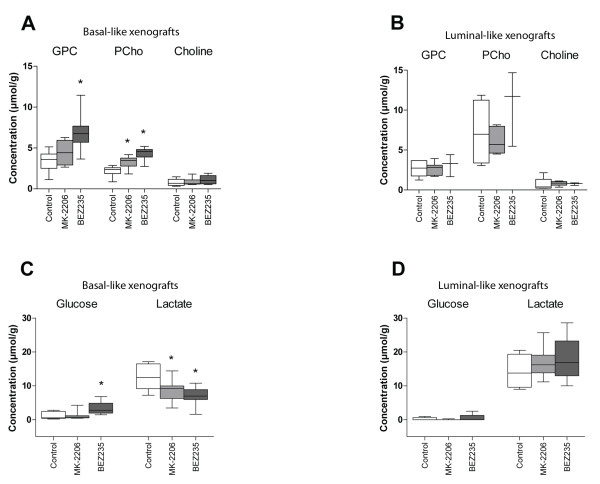
**Phosphatidylinositol 3-kinase inhibition induces changes in glucose and choline metabolism**. **(A), (C) **In basal-like xenografts, treatment with phosphatidylinositol 3-kinase (PI3K) inhibitors increased the concentration of phosphocholine (PCho; both MK-2206 and BEZ235) and glycerophosphocholine (GPC; BEZ235 only). BEZ235 caused an increase in the glucose concentration, whereas both MK-2206 and BEZ235 reduced the lactate concentration. **(B), (D) **No treatment-related changes in these metabolites were observed in luminal-like xenografts. *Significantly different from control (*P *<0.05).

**Figure 7:**
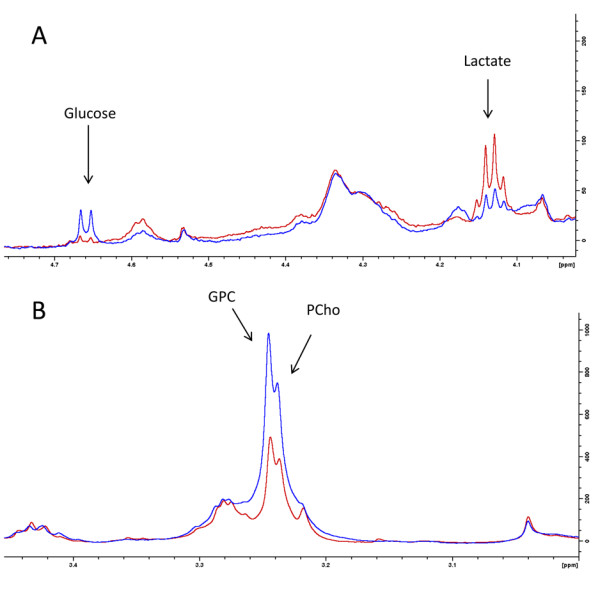
**High-resolution magic angle spinning magnetic resonance spectroscopy demonstrates changes **in **glucose and choline metabolism**. Example spectra from basal-like xenografts illustrate changes in **(A) **glucose and **(B) **choline metabolites. BEZ235 treatment (blue spectra) increased the glucose concentration compared with vehicle control (red spectra). A concomitant decrease in lactate concentration was observed. Both glycerophosphocholine (GPC) and phosphocholine (PCho) concentrations were also increased after BEZ235 treatment.

Treatment-related changes in metabolite concentrations were seen in basal-like xenografts, but not luminal-like xenografts (Figure 6). After treatment with MK-2206, PCho increased by 45% compared with vehicle controls whereas lactate decreased by 33%. In xenografts treated with BEZ235, the metabolic response was more pronounced. PCho and GPC concentrations increased twofold, and lactate concentrations were reduced by 44%. In addition, the glucose concentration was increased nearly threefold. The magnitude of change in the metabolic biomarkers was therefore closely associated with the reduction in pAkt^ser473 ^level. Example spectra illustrating the metabolic changes are presented in Figure 7.

## Discussion

In this study, the response to two PI3K inhibitors with different molecular targets was evaluated in two different breast cancer xenograft models. Combined NIR and confocal immunofluorescence imaging was used to evaluate the baseline level of PI3K signaling in the tumors and to determine the pharmacodynamic effects of drugs targeting the PI3K pathway. *Ex vivo *HR MAS MRS was used to identify metabolic biomarkers for response to therapy. Basal-like xenografts had significantly higher pAkt^ser473 ^levels at baseline, but the phosphorylation was significantly reduced after treatment with BEZ235 and MK-2206. This response was accompanied by early changes in phospholipid and glucose metabolism, reflecting the long-term tumor growth delay caused by PI3K inhibition in this model.

The basal-like and luminal-like xenograft models are established from human primary breast carcinomas directly transplanted to immunodeficient mice. They represent breast cancer with poor (basal-like) and good (luminal-like) prognosis, and have retained the gene expression profile and morphology from the primary tumors [18]. Since patient-derived xenografts represent the cellular heterogeneity of human breast cancer, they are considered to be of high clinical relevance [27]. Previous studies have shown that the basal-like xenograft has a triple-negative phenotype, active angiogenesis and a rapid growth rate compared with the hormone-sensitive luminal-like xenograft model [21]. Gene set enrichment analysis has suggested overactivity in the PI3K signaling pathway [18].

Using a flat-bed NIR fluorescence imager, the levels of pAkt^ser473 ^could be assayed with minimal autofluorescence interference. Subtraction of the signal intensity from tissue sections representing the background levels has been shown to allow quantitative measurement of fluorescent probes with high accuracy [28]. This method allowed semiquantitative analysis of the signal intensity originating from the specifically bound antibodies. This was confirmed by western blotting of the same tissue specimens. The NIR imaging method opens for automated, quantitative imaging of PI3K pathway activity in tumor samples. As for immunostaining in general, this NIR imaging approach is highly dependent on the quality of the antibodies and we have not yet identified an anti-pAkt^thr308 ^antibody that can be used for immunostaining.

The resolution of the images (21 μm) was sufficient to determine relatively fine spatial differences in signaling activity and the scan area is sufficiently large to scan a high number of tumor samples at the same time. However, the method depends on the presence of the phosphorous group at serine 473 in Akt that is responsible for kinase activity. This modification has previously been found labile and is lost over time from isolation of the tumor tissue until fixation or freezing [29]. In the present study, the tumor samples were immediately divided into two parts: one-half was immediately snap-frozen in liquid nitrogen, and the other was immediately fixed. In addition, the fixative was injected into the tumors to avoid dephosphorylation of Akt deeper inside the tumor tissue. In the xenograft tissue, the use of anti-mouse secondary antibodies gave rise to a significant signal in tissue with a high content of murine stromal components. However, the feasibility study performed in human BLBC specimens demonstrated that both total Akt and pAkt^ser473 ^levels could be quantified with high specificity without contribution from unspecific binding of the secondary antibodies. In the clinical setting, the method could be useful for determining activity of Akt for stratification of patients to treatment with PI3K inhibitors. The finding that pAkt^ser473 ^is clearly elevated in only one in five cases of BLBC underscores the importance of subgrouping these patients. Using conventional immunohistochemistry, Lopez-Knowles and coworkers found an elevated level of pAkt^ser473 ^in 24% of 258 invasive breast cancer cases [8]. Interestingly, there is a clear correlation between increased pAkt and loss of PTEN (but not with mutations in PIK3CA) in human tumors and breast cancer cell lines [8,24]. *In vitro *sensitivity for the small-molecule inhibitor LY294002 has been shown to correlate with loss of PTEN [24]. Our finding that the pAkt^ser473^-positive and PTEN-negative basal-like xenograft is sensitive towards both MK-2206 and BEZ235 is thus in line with previous *in vitro *observations.

In this study, two different inhibitors of PI3K signaling were evaluated. MK-2206 is an allosteric pan-Akt inhibitor with broad preclinical anti-tumor activity [19]. BEZ235 is a dual PI3K/mTOR inhibitor, which also has broad antiproliferative effects in a wide range of *in vitro *and *in vivo *cancer models [20]. Both drugs are currently in phase I/phase II clinical trials [30]. PIK3CA mutations, loss of PTEN and increased pAkt levels occur frequently in BLBC. PI3K inhibitors have therefore been suggested as a potentially suitable class of drugs for treatment of this patient group [8,17]. BLBC is strongly associated with the triple-negative phenotype, and because no molecularly targeted treatment options exist for this patient group, PI3K inhibitors have been suggested to be of particular benefit [17]. However, several studies have failed to identify a correlation between PIK3CA mutations and response to PI3K inhibition. The importance of PTEN loss as a single predictive biomarker for response is also debatable [31]. Owing to the complex relationships that determine response to treatment, identification of predictive biomarkers is difficult. Functional biomarkers such as pAkt^ser473^, which more directly is linked to signal transduction activity, may therefore have higher predictive specificity. The current lack of predictive biomarkers for response to PI3K inhibitors calls for alternative stratification strategies. One approach is to identify biomarkers that are associated with changes in the cancer cells after initiation of therapy. Since oncogenic signaling directly regulates key metabolic pathways in cancer, identification of metabolic biomarkers for response to therapy could represent a promising alternative [32].

In this study, the effect of PI3K inhibitors was markedly different in basal-like and luminal-like xenografts. In the luminal-like xenografts, no treatment-related effects on tumor volume, cellular proliferation or pAkt^ser473 ^levels were observed. This indicates that PI3K signaling is not the driving force of tumor growth in this model, which is in accordance with its estradiol addiction [21] and the low baseline level of pAkt^ser473^. The lack of pharmacodynamic response was reflected in the absence of metabolic changes seen in the HR MAS MRS data. In contrast, the basal-like xenograft had a high baseline activity in the PI3K pathway and responded strongly to treatment with both MK-2206 and BEZ235. A long-term delay in tumor growth was observed compared with vehicle-treated controls, concurrent with a reduction in mitotic activity. Furthermore, the levels of pAkt^ser473 ^were reduced to very low levels after 3 days of treatment with the PI3K inhibitors. This observation confirms that the drug indeed hits the target in this model, with concurrent effects on cellular proliferation and tumor metabolism. Both the PHH3 assay and the immunofluorescence imaging analysis suggested that BEZ235 had a stronger inhibitory effect than MK-2206 in basal-like xenografts, with a significant correlation between Akt^ser473 ^phosphorylation and mitotic activity. This differential pharmacodynamic effect between the drugs was also reflected in the metabolic profiles. MK-2206 caused increased PCho concentration and reduced lactate concentration. The magnitude of change in these metabolite concentrations was larger in BEZ235-treated xenografts. In addition, GPC and glucose concentrations were significantly increased. The HR MAS MRS data indicated that PCho, GPC, lactate and glucose are potential metabolic biomarkers for response to PI3K inhibitors. These findings are in accordance with previous studies demonstrating that phospholipid and glucose metabolism pathways contain potential metabolic biomarkers for response to molecularly targeted drugs [11,12].

An abnormally high rate of glucose uptake and utilization is seen in most cancers. In contrast to normal cells, cancer cells extract energy from glucose through glycolysis rather than oxidative phosphorylation, even under normoxic conditions [33]. The low ATP yield is compensated by a high metabolic flux [34]. This way, cancer cells can produce energy while conserving carbon for production of proteins and nucleotides. The glycolytic activity is governed by the cellular microenvironment, but is also regulated by oncogenic signaling [35-37]. The regulatory effect of PI3K signaling on glucose metabolism is complex and multilayered, and includes both Akt-mediated induction of glucose transport and hexokinase activity as well as stimulation of glycolytic rate and lactate production mediated by HIF-1 and Myc [14,38]. Blockade of the PI3K/Akt/mTOR signaling axis has been shown to reduce glycolytic rate and lactate production in cancer *in vitro *[39,40]. The high sensitivity and spectral resolution achieved in our study allowed determination of both glucose and lactate concentration *ex vivo*, demonstrating that inhibition of PI3K signaling both increased glucose concentration and reduced lactate concentration. As the lactate concentration can be measured using *in vivo *MRS, this biomarker is interesting with respect to preclinical therapy monitoring [41]. In the clinical setting, it is difficult to measure the lactate concentration in breast cancer due to the interference from lipids in this tissue. Hyperpolarized ^13^C pyruvate may therefore be the best approach for clinical assessment of glucose metabolism using MRS [42].

The oncogenic signaling pathways that regulate glucose metabolism have also been shown to regulate choline metabolism [13,43]. In breast cancer, abnormally high concentrations of choline metabolites are observed both *in vitro *and *in vivo *[44]. High levels of PCho, GPC and choline were initially associated with a high turnover of cell membrane components in rapidly proliferating cells. Later studies indicated that the abnormal choline metabolism in fact is directly linked to malignant transformation [45]. Although the mechanisms are not fully elucidated, accumulating evidence indicates that synthesis and hydrolysis of PtdCho generates mitogenic messenger molecules such as diacylglycerol, phosphatidic acid, arachidonic acid metabolites and PCho itself [46-49]. Abnormal expression of both choline kinase and phospholipases has been associated with development of cancer [44,50]. It is therefore not surprising that interfering with this metabolic system is considered a valuable therapeutic approach. As an example, drugs inhibiting choline kinase have shown promising anti-tumoral effects in preclinical models and have now entered clinical trials [44,51,52]. However, changes in choline metabolites in response to targeted therapy are poorly understood [53].

From *in vitro *studies, cancer aggressiveness has generally been assumed to be associated with high PCho concentration, and response to therapy assumed to be reflected in decreased concentrations of this metabolite [54,55]. However, it is increasingly recognized that GPC may be a relevant biomarker both in breast cancer and other cancers [26,56,57]. Response to targeted therapy may also be associated with increased concentration of PCho and/or GPC [40,58,59]. The use of choline metabolites as metabolic biomarkers for therapy monitoring therefore requires knowledge about both subtype-specific metabolic profiles and the changes associated with various targeted treatments in each distinct subtype. Choline metabolism may respond differentially to targeted treatment *in vitro *and *in vivo*, and this aspect must also be taken into account [60,61]. In this study, both PCho and GPC increased in basal-like xenografts after blockade of the PI3K signaling. Previous *in vitro *studies of PI3K inhibitors in prostate cancer, colon cancer and breast cancer cell lines have suggested a reduced PCho concentration and an increased GPC concentration, whereas *in vivo *studies in glioblastoma xenografts have suggested a decrease in tCho [40,62,63]. However, we anticipate that the metabolic changes depend on the oncogenic signaling abnormalities seen in different cancer subtypes. The basal-like xenograft model has previously been shown to have a distinct metabolic phenotype, which also was found in a corresponding cohort of human breast cancer biopsies [26]. Our data demonstrate a relationship between PI3K/Akt/mTOR signaling and choline metabolism. As the basal-like xenograft is driven by PI3K signaling, and as its distinct metabolic profile may be associated with this signaling activity, the increased PCho and GPC concentrations observed in this study might possibly be unique features of the MAS98.12 basal-like xenograft. Further studies in a larger panel of basal-like xenografts, representing various genetic backgrounds and metabolic profiles, are needed to elucidate these mechanisms and determine whether the metabolic effects are representative for basal-like breast cancer in general. From a clinical perspective, increased PCho and GPC concentration translates into an increase in tCho, which can be assessed *in vivo *using ^1^H MRS. Alternatively, *in vivo *^31^P spectroscopy could be a possible approach for clinical applications, because this method allows spectral resolution of the phosphomonoesters and diesters PCho, phosphoethanolamine, GPC and glycerophosphoethanolamine in clinical magnetic resonance systems [64].

This study indicates that PI3K inhibitors may be of value in treatment of basal-like breast cancer with high pAkt levels and/or PTEN loss. Early metabolic changes reflected the long-term inhibitory effect on tumor growth. Several studies have suggested that PI3K inhibitors must be combined with other targeted drugs or classical chemotherapy in order to induce apoptosis or kill the cancer cells, and this may also be the case in basal-like breast cancer [65]. As choline metabolism generally is more complex and variable than glucose metabolism in terms of response to therapy, one could assume that assessment of the glycolytic rate and downstream metabolites of glucose may be the most universally applicable approach for identifying relevant metabolic biomarkers. On the contrary, choline metabolism is richer in information and could potentially provide prognostic value in addition to use in therapy monitoring. Finally, it is plausible that lack of a metabolic response, or return to the pretreatment metabolic profile, is associated with primary or acquired drug resistance.

## Conclusion

In summary, we have demonstrated that the PI3K signaling inhibitors MK-2206 and BEZ235 inhibited proliferation and inhibited tumor growth in a basal-like xenograft model. The response correlated to the inhibition of Akt^ser473 ^phosphorylation. No response was seen in luminal-like xenografts, which had lower baseline activity in the PI3K pathway. Using *ex vivo *HR MAS MRS, we found that response to PI3K inhibition was associated with reduced lactate concentration and increased concentration of PCho, GPC and glucose. The magnitude of the metabolic response was reflected the inhibition of cancer cell proliferation and the reduction in pAkt ^ser473 ^level. Since only a subset of patients with BLBC display a clearly elevated pAkt^ser473 ^signal, the sensitivity to PI3K inhibitors may be variable. This heterogeneity underscores the need for functional biomarkers that can predict or detect response to treatment. Lactate, PCho and GPC can potentially be imaged noninvasively *in vivo *using MRS, and may therefore be valuable biomarkers for early monitoring of response to PI3K inhibition in basal-like breast cancer.

## Abbreviations

BLBC: basal-like breast cancer; GPC: glycerophosphocholine; HER2: human epidermal growth factor receptor 2; HR MAS: high-resolution magic angle spinning; MRS: magnetic resonance spectroscopy; mTOR: mammalian target of rapamycin; NIR: near-infrared; PBS: phosphate-buffered saline; PCho: phosphocholine; PHH3: phosphohistone H3; PI3K: phosphatidylinositol 3-kinase; PTEN: phosphatase and tensin homolog.

## Competing interests

The authors declare that they have no competing interests.

## Authors' contributions

SAM conceived and designed the study, conducted the *in vivo *experiments, contributed to data collection and interpretation, and led the writing of the manuscript. CGD developed the methodology for immunofluorescence imaging, and performed the immunofluorescence analysis and the immunoblotting. SSG performed the HR MAS MRS analysis and analyzed the data. AK conducted *in vivo *experiments, and collected and analyzed data. AB supervised the histopathology analysis. GMM and OE established the xenograft models, and participated in the study design and preparation of the manuscript. ISG contributed to study design and supervised data analysis. GB supervised the studies, performed confocal microscopy, interpreted the data and contributed to the preparation of the manuscript. All authors participated in drafting and critically revising the manuscript. All authors read and approved the final manuscript.

## Supplementary Material

Additional file 1 **Table S1 presenting metabolite concentrations of alanine, creatine, choline, phosphocholine, glycerophosphocholine, taurine, glycine, glucose and lactate in basal-like and luminal-like xenografts treated with vehicle, MK-2206 or BEZ235 (µmol/g, mean ± standard deviation)**. *Significantly different from vehicle-treated controls. Click here for file
