# Supplementary material for: Metabolic biomarkers for response to PI3K inhibition in basal-like breast cancer
Source: Breast Cancer Res. 2013 Feb 28;15(1):R16. doi: 10.1186/bcr3391 (PMC3672699; doi:10.1186/bcr3391)
Supplement: Additional file 1 — Table S1 presenting metabolite concentrations of alanine, creatine, choline, phosphocholine, glycerophosphocholine, taurine, glycine, glucose and lactate in basal-like and luminal-like xenografts treated with vehicle, MK-2206 or BEZ235 (µmol/g, mean ± standard deviation). *Significantly different from vehicle-treated controls. [file bcr3391-S1.DOC]

|  | **Basal-like** | | | **Luminal-like** | | |
| --- | --- | --- | --- | --- | --- | --- |
|  | **Vehicle control (n=8)** | **MK-2206**  **(n=7)** | **BEZ235**  **(n=8)** | **Vehicle control (n=6)** | **MK-2206**  **(n=7)** | **BEZ235**  **(n=4)** |
| Alanine | 2.8±1.5 | 2.0±1.2 | 3.0±1.9 | 3.2±1.3 | 3.8±0.9 | 2.8±1.7 |
| Creatine | 1.3±0.8 | 1.7±0.3 | 1.7±0.5 | 2.7±0.9 | 2.0±0.6 | 2.6±0.9 |
| Choline | 0.8±0.4 | 0.6±0.6 | 1.1±0.5 | 0.4±0.2 | 0.8±0.3 | 0.8±0.6 |
| PCho | 2.2±0.6 | 3.2±0.8* | 4.3±0.8* | 7.2±3.5 | 6.0±1.5 | 10.6±4.7 |
| GPC | 3.4±1.3 | 4.4±1.4 | 6.9±2.3* | 2.7±0.9 | 2.7±0.8 | 3.1±1.4 |
| Taurine | 4.8±1.9 | 5.2±1.7 | 4.7±2.3 | 14.0±4.3 | 11.4±2.2 | 10.3±3.4 |
| Glycine | 5.8±2.4 | 5.6±2.0 | 5.7±1.7 | 1.9±0.8 | 2.8±0.8 | 2.4±0.3 |
| Glucose | 1.2±1.1 | 1.3±1.3 | 3.4±1.9* | 0.2±0.4 | 0.04±0.1 | 0.6±1.3 |
| Lactate | 12.6±3.9 | 8.5±3.5* | 7.0±2.8* | 14.2±4.5 | 17.0±4.6 | 18.1±7.8 |

Additional file 1, Table S1: HR MAS MRS can identify biomarkers for response to PI3K inhibition. Metabolite concentrations in basal-like and luminal-like xenografts treated with vehicle, MK-2206 or BEZ235 (µmol/g, mean ±SD). * significantly different from vehicle-treated controls.
